# Response to: Is the new ASNM intraoperative neuromonitoring supervision “guideline” a trustworthy guideline? A commentary

**DOI:** 10.1007/s10877-019-00288-x

**Published:** 2019-02-26

**Authors:** Jeffrey H. Gertsch, Joseph J. Moreira, George R. Lee, John D. Hastings, Eva Ritzl, Matthew Allan Eccher, Jay L. Shils, Gene K. Balzer, Jeffrey R. Balzer, Willy Boucharel, Lanjun Guo, Leah L. Hanson, Laura B. Hemmer, Faisal R. Jahangiri, Jorge A. Mendez Vigil, Richard W. Vogel, Lawrence R. Wierzbowski, W. Bryan Wilent, James S. Zuccaro, Charles D. Yingling

**Affiliations:** 1 Department of Neurosciences, University of California San Diego School of Medicine, La Jolla, CA USA; 2 New York University, Winthrop University Hospital, Mineola, NY USA; 3 Department of Neurology, Vanderbilt University Medical Center, Nashville, TN USA; 4 Aeromedical Neurology, Jacksonville, FL USA; 5 Johns Hopkins University, Baltimore, MD USA; 6 Case Western Reserve University, Cleveland, OH USA; 7 Rush University Medical Center, Chicago, IL USA; 8 Real Time Neuromonitoring Associates, Nashville, TN USA; 9 University of Pittsburgh Medical Center, Pittsburgh, PA USA; 10 Children’s Hospital Colorado, Aurora, CO USA; 11 University of California San Francisco, San Francisco, CA USA; 12 Rhythmlink International, Columbia, SC USA; 13 Northwestern University Feinberg School of Medicine, Chicago, IL USA; 14 AXIS Neuromonitoring, Richardson, TX USA; 15 SpecialtyCare, Brentwood, TN USA; 16 SafePassage Neuromonitoring, New York, NY USA; 17 Avatrode, Bryn Mawr, PA USA; 18 Orlando Health, Orlando, FL USA; 19 Stanford University, and Golden Gate Neuromonitoring, San Francisco, CA USA

On behalf of the membership of the American Society of Neurophysiological Monitoring (ASNM), we welcome our esteemed colleagues’ comments on this new supervision guideline for Intraoperative Neurophysiological Monitoring (IONM) [1, 2]. As with the previous version of this living document, thoughtful comment and criticism assisted the society in crafting this revision and will guide future iterations as well. We applaud Skinner et al.’s pursuit of important concepts such as teambuilding, collaboration, and effective communication. Many of these arguments and aspirations resonate for the authors, even if we do not arrive at the same conclusions. Indeed, revision of the guideline in this instance was triggered by substantive criticism of the previous version’s overreaching aspirational approach to IONM untethered from practical application. The reasonable application of evidence-based outcome data would appear to be an excellent arbiter for most areas of controversy in medicine, if only there were enough material to shed light on some of the more controversial issues (a topic we will return to later).

The crux of Skinner et al.’s concerns appears to revolve around the use of telemedicine in IONM as opposed to personal in-room care, and conceptual arguments from various allied fields of medicine are referenced in support. First and foremost we emphasize that this guideline is not theory—it was written to provide a practical framework for operating in today’s complex medical environment. A guideline establishing a rationale for patient care which may be provided in person, via telemedicine, or some combination of the two, will likely always present challenges in synchronizing standards and conventions. Indeed, even Skinner et al. were obliged to direct their commentary towards a subset of remote providers as opposed to the whole of the field (see Skinner et al.’s footnote 1). They do not however address the thornier practical issues associated with providing care on a daily basis to thousands of patients spread across the United States in hundreds of hospitals with varying sophistication. That a relatively small and potentially declining number of professional IONM subspecialty providers are given the daunting task of providing coverage for a large and continually expanding case volume only highlights the glaring statistical conundrum: there are simply not enough competent providers to cover all of the sites! This important point was not discussed by Skinner et al.

Qaseem et al. would appear to agree; they state on behalf of the Guidelines International Network that “[guideline] recommendations should be supported by careful consideration of evidence; quantification of the magnitude of benefits and harms, as well as costs when possible; resource and feasibility issues; implementation considerations; patient and caregiver preferences and concerns; and ethical and legal matters” [3]. Which is to say, medicine is not practiced in a vacuum; rather in a complex environment with a myriad of considerations that must constantly be re-balanced in real time. When the IONM professional is faced with the real-world challenges associated with providing care for multiple simultaneous patients in different hospitals, choices must be made as to the best use of resources to provide the safest, most principled and competent care to which Skinner et al. aspire. Which case should I attend in person? Should care be denied for one in preference of another (rationing?), and if not then what choice is there today but to utilize a telemedicine solution? What FDA-approved telemedicine solutions are available now and what are their technological limitations? If I use telemedicine, then how many simultaneous cases—each with varying complexity—is too many? If the complexity equation changes with these multiple cases (i.e. there is an emergency), what is the availability of my backup given this similarly busy subspecialist does not simply wait in the wings for intermittent disaster to strike? These are difficult, real world questions but as guideline authors we do not dictate in specific—we guide the well-intended professional according to rational principles supported by the best available evidence. That the evidence base on practice patterns is extremely limited is fully recognized, so we fill gaps with expert consensus. This strategy did not invalidate the initial guideline, nor does it invalidate its revision either.

Skinner et al. level oblique criticism towards the guideline authors as protecting commercial interests; such criticisms are unfounded and the authors vigorously assert their independence. Skinner et al. should clarify and direct such claims more explicitly towards the offending commercial sector in a separate editorial as opposed to broadly criticizing a professional guideline and cast aspersions on the entire IONM field. This ASNM practice guideline does not in any way protect, endorse, or support unethical or unprofessional conduct in any aspect of IONM. We reiterate that professionals are provided with guiding principles and allowed at their discretion to determine the best method of care for an individual patient given the resources available. It is most important for any IONM delivery model that all personnel be highly educated in the practice of IONM and diligent in their roles; the specific model is not as important as the manner in which care is communicated and delivered. The revised ASNM guideline ensures IONM professionals are truly competent and qualified to practice, regardless of their model, by stating “Board certification relevant to the practice of IONM patient care remains necessary to practice as an IONM-P” [1].

Lastly and most important, the revision guideline authors wish to make explicit mention of the current woeful status of the evidence base in IONM supervision paradigms. We are not immune to many of the well-intentioned arguments Skinner et al. advance. That said, this is twenty-first century medicine and there are minimal outcomes data to elevate in-room supervision paradigms over live remote supervision/communication, a well-known if exasperating shortcoming in the medical literature. We call upon Skinner et al. to join with guideline authors and undertake the research needed to generate evidence for in-room versus remote IONM practice paradigms that can be critically evaluated and incorporated into the next version of the guideline. As little actual data exist, the guideline will stand until such time as paradigm-changing information is available. In the meantime, the authors assert that all patients, regardless of their proximity to a neurophysiologist, deserve access to the protections afforded by IONM.
